# Safety and Feasibility of Endoscopic-Assisted Electrochemotherapy in Addition to Neoadjuvant Treatment of Locally Advanced Rectal Cancer: An Exploratory Randomised Clinical Trial †

**DOI:** 10.3390/cancers18183066

**Published:** 2026-09-21

**Authors:** Loan Ngo-Stuyt, Rasmus Peuliche Vogelsang, Mustafa Bulut, Helle Hjorth Johannesen, Annika Loft, Malene Broholm, Mikail Gögenur, Adile Orhan, Michael Seiersen, Trine Stigaard, Julie Gehl, Ismail Gögenur

**Affiliations:** 1 Center for Surgical Science, Department of Surgery, Zealand University Hospital, Lykkebækvej 1, 4600 Køge, Denmark; rvo@regionsjaelland.dk (R.P.V.); mgog@regionsjaelland.dk (M.G.); aor@regionsjaelland.dk (A.O.); mics@regionsjaelland.dk (M.S.); igo@regionsjaelland.dk (I.G.); 2 Department of Clinical Medicine, University of Copenhagen, Blegdamsvej 3B, 2200 Copenhagen, Denmark; kgeh@regionsjaelland.dk; 3 Copenhagen Academy for Medical Education and Simulation (CAMES), University of Copenhagen and the Capital Region of Denmark, Ryesgade 53B, 2100 Copenhagen, Denmark; 4 Department of Clinical Physiology, Nuclear Medicine and PET, Rigshospitalet, Copenhagen University Hospital, Blegdamsvej 9, 2100 Copenhagen, Denmark; helle.hjorth.johannesen.01@regionh.dk (H.H.J.); annika.loft@regionh.dk (A.L.); 5 Department of Infectious Diseases, Amager and Hvidovre Hospital, Kettegård Alle 36, 2650 Hvidovre, Denmark; malene.broholm.andersen@regionh.dk; 6 Department of Clinical Oncology, Zealand University Hospital, Sygehusvej 10, 4000 Roskilde, Denmark

**Keywords:** electrochemotherapy, electroporation, colorectal neoplasms, neoadjuvant therapy

## Abstract

Background: Locally advanced rectal cancer (LARC) has high recurrence rates despite aggressive multimodal treatment with neoadjuvant chemoradiotherapy (CRT) followed by surgery. Electrochemotherapy (ECT), combining electroporation with chemotherapy, has shown safety and feasibility in other malignancies, but its role as an additive neoadjuvant treatment in rectal cancer remains unexplored. Methods: In this parallel-group, randomised safety and feasibility trial, patients with histologically verified LARC who completed neoadjuvant CRT were randomised 1:1 to a single ECT treatment with bleomycin before surgery (*n* = 8) or standard therapy (*n* = 8). The primary outcome was safety (amended from the protocol-specified efficacy endpoint after early study termination). Follow-up was scheduled before surgery and at 6 and 12 weeks post-surgery, including adverse events, clinical examination, and patient-reported quality of life, bowel, urinary, and sexual function. Results: ECT was successfully delivered in all treated patients. Median procedure time was 23.5 min. Adverse events occurred in all ECT patients, predominantly rectal pain (86%), with one patient hospitalised for pain management. No procedure-related complications were observed. Two ECT patients had R1 resections due to intraoperative perforations. Tumour response was heterogeneous: two patients achieved complete response (TRG1), three achieved partial response (TRG2–3), and one achieved no response; corresponding numbers in the control group were 2, 4, and 2. One ECT patient achieved clinical complete response and entered a watch-and-wait programme. Patient-reported pain worsened transiently after ECT but returned to baseline by 12 weeks post-surgery. Conclusions: ECT is a technically feasible adjunct to neoadjuvant CRT for LARC. However, its efficacy and safety, particularly regarding surgical complications, remain unclear. Larger trials are needed to determine the role of ECT in multimodal neoadjuvant treatment.

## Simple Summary

This randomised controlled trial investigated the feasibility and safety of adding electrochemotherapy (ECT) before surgery in patients with locally advanced rectal cancer as an add-on to standard neoadjuvant chemoradiotherapy. Patients were randomised to receive either a single ECT treatment with bleomycin prior to surgery or standard care alone. The aim was to assess safety, as well as treatment response and patient-reported outcomes. All ECT treatments were successfully completed, with a median procedure time of approximately 24 min. Adverse events were common after ECT, most frequently rectal pain, and one patient required hospital admission for pain control. No direct procedural complications were reported, although two patients in the ECT group had R1 resections due to intraoperative perforations. Overall, ECT was technically feasible to deliver in this setting, but its safety and effectiveness require further evaluation in larger studies.

## 1. Introduction

Colorectal cancer (CRC) is one of the most commonly diagnosed cancers in the world, with nearly 2 million new cases diagnosed worldwide annually [1,2]. Rectal cancer accounts for around one third of all CRC cases but is associated with inferior oncological outcomes, including lower disease-free and overall survival rates compared to colon cancer [1,3]. Conventional management for locally advanced rectal cancer (LARC) involves a multimodal approach, typically comprising neoadjuvant chemoradiotherapy (CRT) followed by surgical resection with total mesorectal excision [4]. Clinical complete response (cCR) following neoadjuvant CRT is achieved in approximately 20% of patients and is associated with better oncological outcomes [5]. However, despite this aggressive treatment approach, up to 30% of LARC patients develop local or distant recurrence [6,7]. Moreover, the intense multimodality treatment is associated with high morbidity and impaired quality of life, both during and after completed treatment [8,9,10].

Electroporation is a non-invasive ablation treatment, utilising short-voltage pulses to produce a transient increase in cell membrane permeability [11]. The formation of pores across the phospholipid bilayer allows the passage of otherwise poorly or non-permeating substances such as hydrophilic chemotherapeutics [12]. The first use of electrochemotherapy (ECT) in oncology occurred almost 4 decades ago [13,14], and has since been established as a safe and effective therapy in the treatment of cutaneous and subcutaneous tumours [15,16] with complete response rates of up to 70 per cent reported [16]. There is a growing interest in the use of electroporation in solid tumours. Early preclinical and clinical experiences from liver, kidney, pancreas, colorectal, oesophageal, vulvar, and head and neck cancer have demonstrated electroporation as a feasible treatment with an acceptable safety profile [17,18,19,20,21,22,23,24,25,26]. The first-in-human trial of ECT in inoperable CRC patients demonstrated clinical efficacy without serious adverse events during the procedure or in the postoperative period [17]. However, the safety of ECT in the treatment of non-metastatic CRC has not been established, as data on adverse effects are limited.

The aim of this randomised safety and feasibility clinical trial was to investigate ECT as an additive treatment to the standard neoadjuvant down-staging treatment in LARC patients prior to curative-intent surgery.

## 2. Materials and Methods

### 2.1. Trial Design and Oversight

This was a parallel-group, randomised safety and feasibility clinical multicentre trial investigating neoadjuvant ECT in LARC patients as part of an additive down-staging treatment prior to curative-intent surgery. In addition, the efficacy of ECT as an add-on treatment to standard neoadjuvant CRT was explored. Overall, 40 patients were planned to be included in the trial: 20 patients in the ECT group and 20 patients in the control group. Patients were recruited between January 2017 and February 2019 at Zealand University Hospital, Copenhagen University Hospital Herlev and Gentofte, and Slagelse Hospital, Denmark. Patients were randomised 1:1 to either the intervention group treated with one series of ECT treatment with bleomycin prior to surgical resection or the control group with standard therapy. The protocol was approved by the Danish Medicines Agency, EudraCT nr. 2016-00015-40, the Regional Ethics Committees, SJ-518, and the Danish Data Protection Agency, REG-032-2016 (approval date 13 May 2016). The study was registered prior to patient inclusion at Clinicaltrials.gov (NCT03040180) https://clinicaltrials.gov/study/NCT03040180?term=NCT03040180&viewType=Card&rank=1&tab=study&a=2 (accessed on 26 May 2026). Trial data were externally monitored by the Good Clinical Practice unit at Copenhagen University Hospital, Frederiksberg Hospital, Denmark. The trial was prematurely terminated before reaching the planned sample size due to recruitment challenges arising from the logistical complexity of the multi-site design, which required participating patients to visit multiple hospitals, in combination with resource constraints and the impact of the COVID-19 pandemic.

### 2.2. Patients

Eligible patients had histologically verified, locally advanced rectal cancer, UICC stage II or III. All cases had been reviewed and deemed curable by a multidisciplinary team (MDT) consisting of specialists in colorectal surgery, oncology, radiology and pathology. All patients had completed the standard of care neoadjuvant CRT prior to study enrolment. Patient inclusion criteria were age ≥ 18 years, ASA classification ≤ 3, and patients deemed capable of understanding the given information. Exclusion criteria were coagulation disorders, highly inflamed gastrointestinal tissue with ulceration and bleeding, implantable cardioverter defibrillator (ICD) or pacemaker, epilepsy, hepatitis B or C, HIV infection, concurrent treatment with phenytoin, clozapine or an investigational medicinal product, pregnancy or lactation in women, patients who had undergone treatment with bevacizumab within 4 weeks prior to enrolment, contraindications for positron emission tomography/magnetic resonance imaging (PET-MRI) or bleomycin, and registration in the National Database of Non-Consent to the Use of Tissue Samples for Scientific Purposes. All patients provided written informed consent.

**Figure 1:**
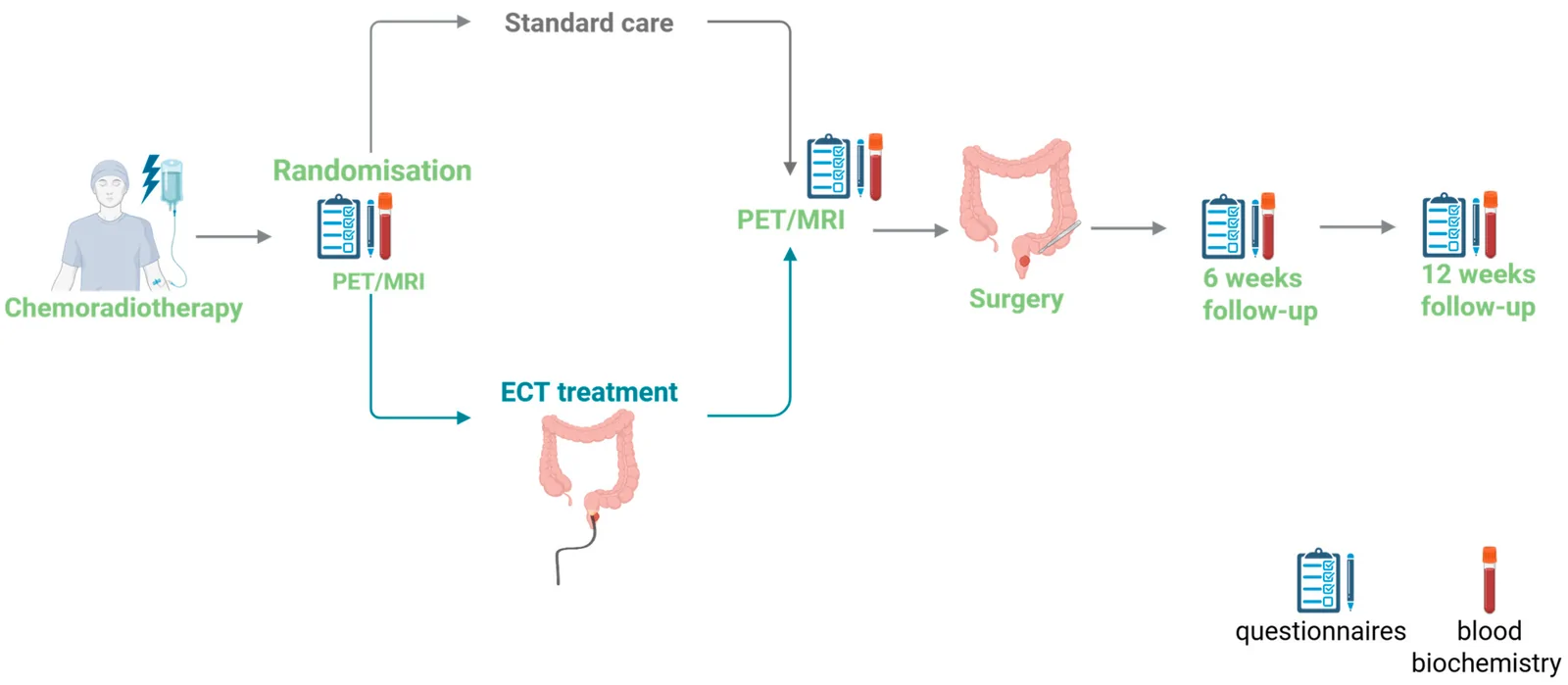
Study design. Created in BioRender. Ngo-Stuyt, L. (2026) https://BioRender.com/mkpbdtd (accessed on 26 May 2026).

All patients underwent standard CRT, which consisted of long-course radiotherapy delivered at a total dose of 50.4 Gy (28 fractions × 1.8 Gy) each with concomitant capecitabine (1650 mg/m^2^) prior to inclusion. Patients were included 1–2 weeks after completion of CRT. The decision to include patients after completion of CRT was made to avoid potential adverse events related to ECT compromising the delivery of neoadjuvant CRT. Prior to randomisation, all patients underwent a clinical examination, blood biochemistry, electrocardiography (ECG), and filled out questionnaires assessing quality of life (QoL), bowel function, urinary tract symptoms, and sexual function. A PET-MRI was performed in the weeks leading up to surgical resection as a measure of response to the neoadjuvant ECT treatment. Blood biochemistry and questionnaires were also assessed prior to surgery. Follow-up visits scheduled at 6 weeks and 12 weeks after surgery consisted of clinical examination, blood biochemistry and questionnaires. Figure 1 depicts the trial design.

### 2.3. Electrochemotherapy Procedure

Patients randomised to intervention were treated with a single ECT session 3–5 weeks after completion of standard neoadjuvant CRT. The procedure was performed under general anaesthesia or nurse-assisted propofol sedation (NAPS). A linear endoscopic vacuum probe (EndoVe, Mirai Medical Ltd., Unit 5 Howley Court, Oranmore, Galway, Ireland) developed for delivery of Pulsed Electric Fields (PEF) in the gastrointestinal tract was used in all ECT procedures. Cliniporator EPS01 (IGEA Ltd., 41012 Via Parmenide, Carpi, Italy), a square wave pulse generator, was used for pulse generation. Cliniporator delivered a series of eight pulses of 0.1 ms duration with a frequency of 1 Hz and amplitude of 1 kV/cm (electrode distance). Prior to electroporation, a bleomycin infusion of 15,000 IU/m^2^ body surface area was given. The electroporation procedure was initiated 8 min after injection of bleomycin, according to the ESOPE guidelines [27]. The tumour tissue was drawn under a vacuum between 50 and 400 mmHg, after which PEF was applied. Pulse duration was in the range of 100 µs. If necessary, the electrode was repositioned to ensure treatment of the entire tumour. All ECT procedures were performed by two board-certified surgical endoscopists.

### 2.4. Outcomes

#### 2.4.1. Primary Outcome

The protocol-specified primary outcome was treatment efficacy, assessed by pathological examination of the resection specimen following surgery using the five-tiered Mandard tumour regression grade (TRG) scale. The study was terminated prematurely due to failure to recruit patients at an acceptable rate, with only 16 patients enrolled compared with the planned 40. Given the resulting reduced sample size, the study became insufficiently powered to meaningfully assess efficacy as a primary outcome. The primary outcome was redefined as treatment safety, assessed by adverse events (AEs) according to the Common Terminology Criteria for Adverse Events (CTCAE) scale version 4.0, which had originally been protocol-specified as a secondary outcome. Although the study was not adequately powered to assess the safety outcome, safety could still be systematically and descriptively assessed in the reduced cohort, whereas the small sample size made a comparative assessment of efficacy unreliable.

#### 2.4.2. Secondary Outcomes

##### Surgical Complications

Treatment safety of surgery following ECT was assessed by the number of patients with compromised surgical outcomes after the intervention, defined as an R1 resection.

##### Postoperative Complications

Postoperative complications were registered and classified according to the Clavien–Dindo classification.

##### Efficacy

Efficacy was measured by tumour response following ECT, assessed by histopathological evaluation of TRG. Tumour response was also explored using PET-MRI, measured by changes in SUVmax and tumour volumetrics.

##### Tumour Immunologic Response

Tumour immunologic infiltration following ECT assessed through translational analyses is also planned and will be reported separately and is not included in the present analysis.

#### 2.4.3. Patient-Reported Outcome Measures

Patient-reported outcomes were assessed using validated Danish questionnaires covering bowel, urinary, and sexual function, pain, as well as overall quality of life at four time points: baseline, before surgery, 6 weeks and 12 weeks post-surgery. The European Organisation for Research and Treatment of Cancer Quality of Life Questionnaire Core 30 (EORTC QLQ-C30) [28] is a 30-item core questionnaire designed to capture the overall QoL of cancer patients across functional scales (physical, role, emotional, cognitive, social) and several symptom scales and items, including pain, fatigue and gastrointestinal symptoms. Pain was assessed using the 11-point Pain Intensity Numeric Rating Scale (NRS) [29], where 0 indicates no pain, and 10 indicates the worst possible pain. The questionnaire evaluates pain across three domains: current pain, worst pain and average pain during the last 7 days. Bowel function was assessed using the Low Anterior Resection Syndrome (LARS) score [30] developed to capture anorectal dysfunction related to incontinence for flatus and liquid stools, frequency of bowel movements, clustering of stools, and urgency. The patients are sub-grouped according to the total score into no LARS, minor LARS, and major LARS. Only major LARS is associated with a marked reduction in QoL [31]. The International Consultation on Incontinence Questionnaire Male Lower Urinary Tract Symptoms Module (ICIQ-MLUTS) [32] and Female Lower Urinary Tract Symptoms Module (ICIQ-FLUTS) [33] are questionnaires that include domains of voiding, incontinence, and frequency in men and women, respectively. The International Index of Erectile Function (IIEF) [34] is a 19-item questionnaire assessing male sexual function across five domains: erectile function, orgasmic function, sexual desire, intercourse satisfaction, and overall satisfaction. For female sexual function, the Sexual Function Vaginal Changes (SVQ) [35] questionnaire was used, assessing intimacy, sexual interest, sexual satisfaction, vaginal changes, and sexual functioning.

### 2.5. Image Acquisition

Study participants met after 4 h of fasting at the Department of Clinical Physiology and Nuclear Medicine, Rigshospitalet, and were scanned using an integrated PET-MRI system (Siemens Biograph mMR, Siemens Medical Solutions, Malvern, PA, USA). For the PET scan, an intravenous dose of 4 MBq/kg body weight of 18F flouro-deoxy-glucose (FDG) was injected 60 min prior to scanning. A whole-body PET scan was obtained simultaneously with the MRI scan. A single dose of 1 mL Buscopan 20 mg/mL (Boehringer Ingelheim, Barcelona, Spain) was administered as an intramuscular injection immediately prior to scanning to avoid motion artefacts due to intestinal motility. One pelvic bed position was scanned: Vendor’s standard T1-weighted two-point Dixon MRI sequences were acquired for PET attenuation correction; T2-weighted sequences in transversal, sagittal, paracoronal and paratransversal were acquired for tumour delineation; the transversal T2 sequence with a slice thickness of 4 mm, the others with 3 mm slice thickness. In addition, diffusion-weighted MRI sequences with b-values of 0 s/mm^2^ and 800 s/mm^2^ were performed, and the apparent diffusion coefficient (ADC) was calculated. The volumetric analysis of the T2 sequences was performed using the software Mirada RTx version 1.6.7.2 (MIRADA Medical, Oxford, UK), delineating the tumour on the paratransversal sequence supported visually by the other T2w images and the ADC maps. The fused PET-MRI images were interpreted by a specialist in nuclear medicine and a specialist in diagnostic radiology side-by-side with measurements of tumour volume and metabolic activity by Standardised Uptake Value (SUVmax).

### 2.6. Randomisation

The randomisation of patients was conducted using prefabricated and sequentially numbered, sealed, opaque envelopes. Each envelope contained a number with a corresponding Case Report Form (CRF) and a randomly assigned treatment regimen.

### 2.7. Blinding

The assessing radiologist, nuclear medicine physician and pathologists assessing MRI, PET and tumour regression grade, respectively, were blinded in the trial. Patients and the treating clinical personnel were not blinded.

### 2.8. Statistical Analysis

This study is the first to assess neoadjuvant ECT treatment in LARC. Based on previous exploratory preclinical studies, it was determined that a sample size of 40 patients was appropriate. With 20 patients per group, the study was expected to detect a 50% increase in the proportion of patients achieving tumour regression grade 2 or higher in the ECT plus CRT group compared with the conventional treatment group, with a type 1 error of 5% and power of 80%. This power calculation pertained to the original efficacy primary outcome; following early termination and the re-designation of safety as the primary outcome, no formal sample size calculation was performed for the safety endpoint, and all analyses are therefore descriptive. Descriptive analyses were performed according to the randomised treatment allocation (intention-to-treat principle). However, efficacy outcomes based on resected specimen and PET-MRI were only available in subsets of patients, and analyses were therefore conducted in available evaluable populations and should be considered exploratory. The safety population included all patients who received the allocated treatment and for whom safety data were available. AEs were assessed from the time of treatment until after surgery. The pathology-evaluable population consisted of all patients who underwent surgical resection. The imaging-evaluable population consisted of patients with PET-MRI scans available at both baseline and pre-surgery. Tumour response was calculated and plotted individually for each study participant. Missing data in the patient-reported outcome measures were handled using prorated imputation when at least half of the items in the domain were completed. Domains with fewer than 50% of the items answered were treated as missing. Non-parametric statistical testing using the Wilcoxon rank-sum test was performed where appropriate due to the small sample size. Statistical analyses were performed using R software version 4.3.0.

Reporting of this study was done in accordance with the CONSORT guidelines for reporting of randomised trials.

## 3. Results

### 3.1. Participant Flow

**Figure 2:**
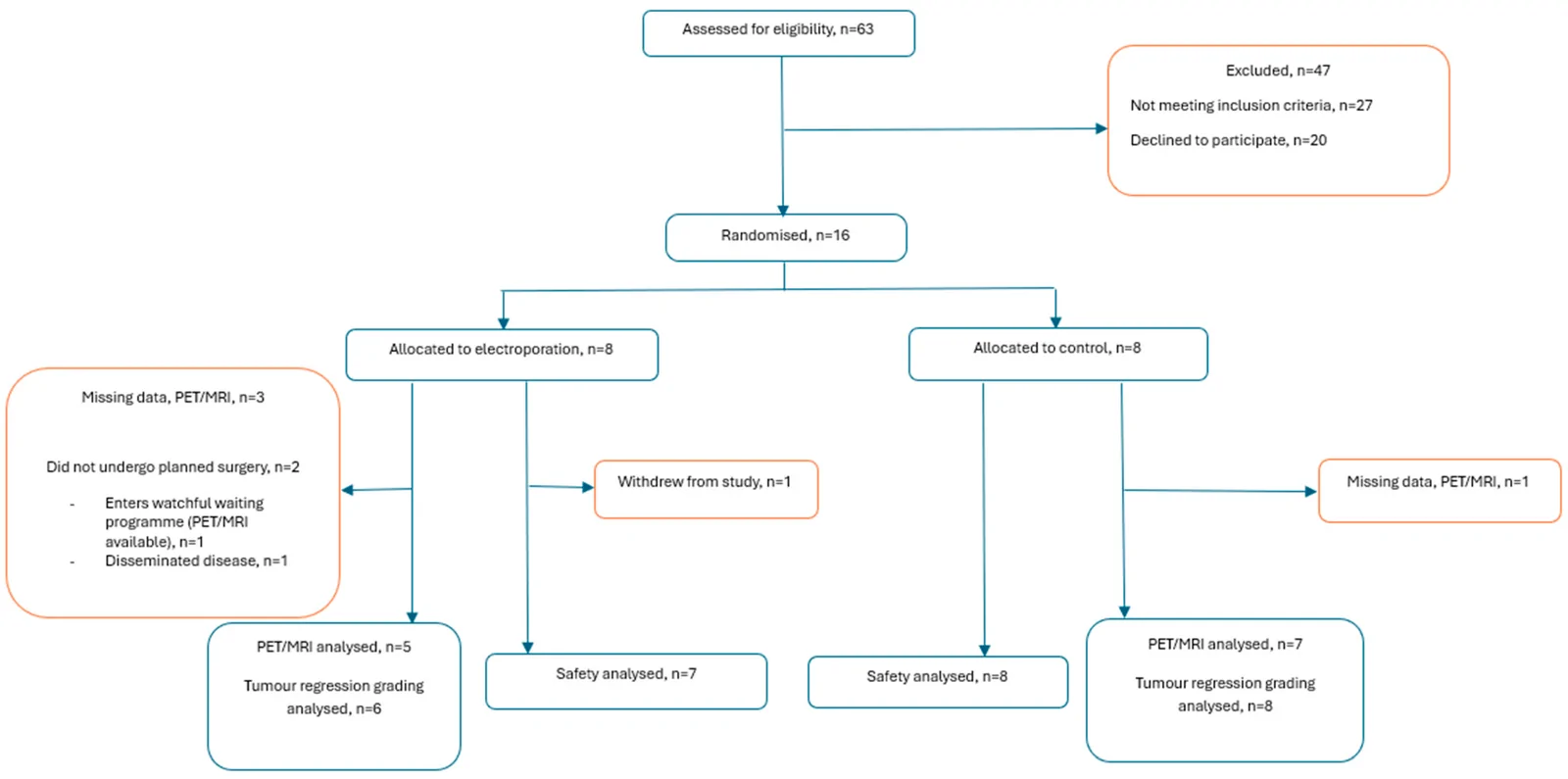
Participant flow.

A total of 63 patients were screened between January 2017 and February 2019, of whom 47 were excluded. Patient enrolment in the trial was prematurely terminated due to failure to recruit patients at an acceptable rate. The 16 patients included in the trial were randomised equally to the ECT group (*n* = 8) and the control group (*n* = 8). In the ECT group, three patients had incomplete PET-MRI data, and two did not undergo the planned surgical resection after ECT treatment. In the control group, one patient had incomplete PET-MRI data. For the safety analysis, all patients were included in the control group, while one patient in the ECT group withdrew from the study. Participant flow is depicted in Figure 2.

### 3.2. Baseline Data

Patient characteristics are shown in Table 1. Ten men and 6 women were included, with an overall median age of 59 (IQR 50–67). The majority of patients had T3 cancer (81%) with lymph node involvement. The control group consisted of a higher number of UICC 3 patients and patients who scored higher on the American Society of Anaesthesiologists (ASA) physical status classification. Adenocarcinoma (81%) was the most prevalent primary histological diagnosis overall, followed by mucinous adenocarcinoma (13%) and glandular carcinoma (6.3%). Prior to inclusion in the study, all patients underwent CRT, which consisted of radiotherapy delivered at a total dose of 50.4 Gy in 28 fractions of 1.8 Gy each. Concurrent capecitabine was administered at a median dose of 1650 mg (range 1300–1950). Additionally, one control patient received oxaliplatin at a dose of 150 mg while another control patient received panitumumab at a dose of 400 mg. One ECT patient received oxaliplatin at a dose of 240 mg. Oxaliplatin was added in both cases due to a pulmonary infiltrate suspicious for malignancy. Panitumumab was added due to a KRAS wild-type status.

### 3.3. Electrochemotherapy Procedure

All procedures were initiated with endoscopic inspection of the tumour area. Subsequently, bleomycin was administered intravenously, with a median dose of 30,000 IU (range 24,000–30,000), prior to electroporation of the tumour area. The median procedure time was 23.5 min (range 9–36), while the number of delivered electrical pulses was 6 (range 3–15). Complete treatment of the tumour surface was achieved in all patients except one, in whom 75% of the surface was treated. No complications occurred in relation to the ECT procedure. Median time between neoadjuvant CRT completion and ECT was 29 days (range 25–33).

### 3.4. Safety Outcomes—Adverse Events

AEs occurred in all patients in the ECT group (*n* = 7), compared to 50% in the control group (*n* = 4). Safety outcomes and AEs are presented in Supplementary Table S1a,b. A total of 27 AEs or SAEs were registered in 7 ECT patients during the study period. Of these, 21 were electroporation- or bleomycin-related and were observed in 6 patients. Electroporation-related AEs were reported in six (86%) patients, with rectal pain being indisputably the most frequently observed, reported in six out of the seven ECT patients included in the safety analyses. Bleomycin-related AEs involved common side effects such as fever, nausea/vomiting and fatigue, and were recorded in one patient. Three SAEs were registered in two patients. One SAE was electroporation-related, involving a patient admitted to the hospital 20 days after ECT treatment due to rectal pain, which could not be managed with medication. The patient was successfully treated with an ultrasound-guided pudendal nerve block and an epidural block. Two months later, the same patient was admitted to hospital due to fatigue, deemed unrelated to ECT treatment. The second patient suffered a fall which necessitated hospital admission, also unrelated to ECT treatment.

### 3.5. Surgical Resection and Post-Operative Complications

**Figure 3:**
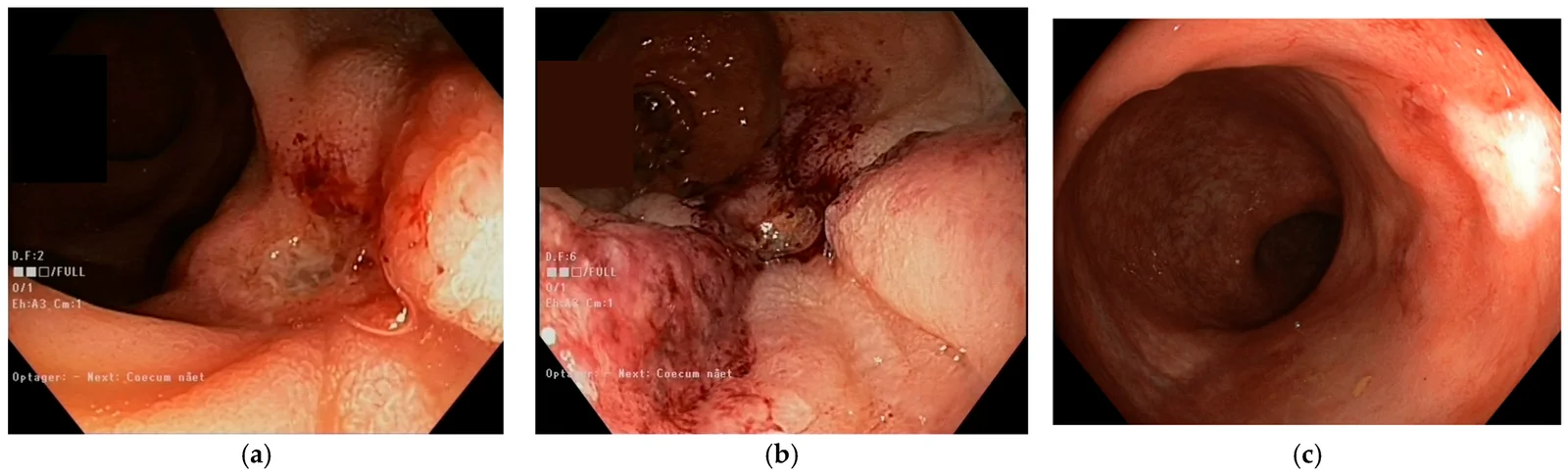
(**a**) Before ECT (after completed neoadjuvant CRT), showing a low rectal tumour. (**b**) Immediately after ECT, showing ischemic tissue in the treated area. (**c**) One year after ECT, showing scar tissue and no residual tumour.

Fourteen out of the 16 patients underwent surgical resection as scheduled. Surgery characteristics are shown in Table 2. In the ECT group (*n* = 6), surgery was performed within a median of 43 days (range 29–46) after ECT treatment. Eleven operations were scheduled as laparoscopic or robotic, while three operations were open. None of the laparoscopic/robotic surgeries in either group was converted to open surgery. Macroscopic R0 resection was not achieved in two cases in the ECT group due to intraoperative perforation. In one case, an anterior perforation occurred during dissection of the anal part in an abdominoperineal resection performed 44 days after ECT. The operating surgeons attributed this to dense fibrotic tissue presumably due to neoadjuvant radiation therapy. In the second case, tumour perforation occurred during a TaTME procedure 43 days post-ECT, with no identifiable contributing factors registered. Pathological examination showed a TRG 4 in the first case and TRG 3 in the second. Postoperative complications were classified according to the Clavien–Dindo classification. Three postoperative complications were observed in two patients in the ECT group, two grade 1 and one grade 3, involving upper gastrointestinal bleeding, urinary retention and paralytic ileus. In the control group, four postoperative complications were observed in three patients, two grade 1 and two grade 3, involving ocular migraine, knee effusion, sepsis, and abscess in the perineal cicatrix. No surgical reinterventions were required. Two patients in the ECT group did not undergo the planned rectal resection. Clinical complete response was achieved in one patient following ECT treatment. After MDT review and a thorough discussion between the patient and surgeon, a watch-and-wait approach was chosen. The patient entered a surveillance programme with regular colonoscopies and remains cancer-free 4.5 years after ECT treatment (Figure 3 depicts the patient’s endoscopy images). In the second case, local tumour regression of the rectal cancer was found. However, the finding of concurrent liver metastases rendered the patient ineligible for the planned surgical course.

### 3.6. Efficacy Outcomes

**Figure 4:**
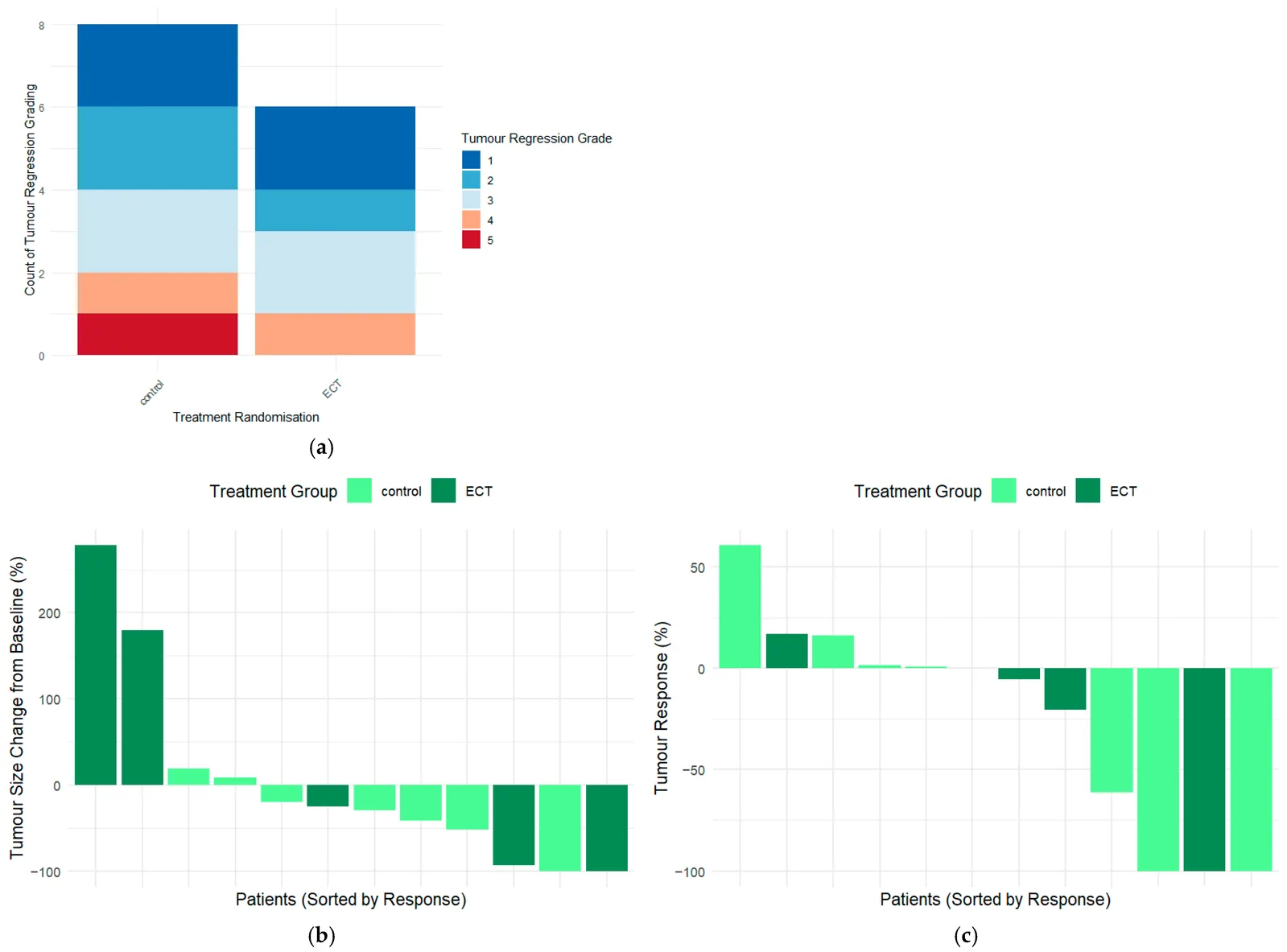
(**a**) Tumour regression grading, Mandard classification. Complete response: 1. Partial response: 2–3. No response: 4–5. (**b**) Waterfall plot of individual tumour volumetric response as measured by MRI. (**c**) Waterfall plot showing individual tumour response on PET, measured by SUVmax.

Pathological tumour regression was assessed using the Mandard classification and depicted in Figure 4a. In the ECT group (*n* = 6), two patients had a complete response (tumour regression grade 1), while three patients (one with grade 2 and two with grade 3) had a partial response. One patient had no response. In the control group (*n* = 8), two patients had a complete response, while four patients demonstrated a partial response. The remaining two patients had no response.

Tumour volumetric responses assessed by MRI (Figure 4b) showed that in the ECT group (*n* = 5), one ECT patient had a complete tumour response, while another had a near-complete response. Additionally, one ECT patient had a partial tumour response, while tumour progression was found in the remaining two patients. In the control group (*n* = 7), one patient had a complete tumour response, four had a partial tumour response, and the remaining two showed tumour progression. Tumour responses assessed by PET (Figure 4c) showed three patients with complete responses, one in the ECT group and two in the control group. Two ECT patients had a partial tumour response, one showed tumour progression, and the last patient had stable disease. Conversely, in the control group, one patient had a partial response, two showed disease progression, and two had stable disease. Median time between completed neoadjuvant CRT and baseline PET-MRI was 15 days (range 13–26), while median time between ECT and the second PET-MRI was 38 days (range 26–42). In the ECT group, PET-MRI data for one patient who did not undergo the planned surgery was available for analysis. Efficacy outcomes measured by MRI, PET and tumour regression grading were grouped together, and long-term recurrence status for each individual patient is presented in Supplementary Table S2a,b.

#### 3.6.1. Quality of Life

The results of the EORTC QLQ C-30 questionnaires are presented as radar charts (Supplementary Figure S1a–d) and summarised in tables (Supplementary Table S3a,b) for both the ECT and control groups. Following ECT treatment and before surgery, patients in the ECT group scored lower on the function scales and higher on the symptom scales, indicating a reduced quality of life following ECT treatment in the intervention group. The most notable differences were observed in the symptom scales and items pain (*p* = 0.0009), fatigue (*p* = 0.001), constipation (*p* = 0.001), and insomnia (*p* = 0.007), as well as in the function scales social functioning (*p* = 0.003), role functioning (*p* = 0.003) and physical functioning (*p* = 0.005), emotional functioning (*p* = 0.009), and global health status (*p* = 0.01). Quality of life was regained in the ECT group by 12 weeks post-surgery, with no statistically significant differences observed between the two groups.

#### 3.6.2. Pain

Pain assessment using the NRS (Supplementary Table S4) showed that patients in the ECT group reported higher pain scores across all three domains at baseline, before surgery, and 6 weeks post-surgery. However, by 12 weeks post-surgery, pain scores in both the control and ECT group appeared similar.

#### 3.6.3. Anorectal Function

Anorectal function was assessed using the LARS score and is presented as boxplots (Supplementary Figure S2). At baseline, after completing CRT, 87.5% (*n* = 7) of patients in the ECT group and 50% (*n* = 4) in the control group had scores corresponding to major LARS. After surgery, no patients in either group had scores corresponding to major LARS. Minor LARS was reported by two patients in each group at 6 weeks post-surgery, decreasing to one patient per group at 12 weeks post-surgery, indicating gradual recovery of anorectal function over time after surgery.

#### 3.6.4. Urinary Function

Urinary function for male patients assessed using the ICIQ-MLUTS questionnaire is presented in linear charts over time (Supplementary Figure S3a). Patients in the ECT group generally reported higher symptom scores across most domains and time points. Overall, urinary function appeared to deteriorate transiently following ECT treatment but improved postoperatively, reaching similar levels to the control group by 12 weeks post-surgery.

ICIQ-FLUTS scores were available for six women (Supplementary Figure S3b), limiting meaningful between-group comparisons. Patients in the control group had consistently lower urinary symptom scores. In the ECT group, median scores for filling, voiding, and incontinence symptoms were higher at baseline and showed more pronounced fluctuations during follow-up.

#### 3.6.5. Sexual Function

Only a very small number of male and female participants completed the sexual function questionnaires, preventing meaningful interpretation of the findings. The available data are presented in Supplementary Tables S5 and S6.

## 4. Discussion

In this early randomised controlled study, we investigated the feasibility, safety, and efficacy of ECT as an additive treatment to standard neoadjuvant CRT in LARC patients prior to curative-intent surgical resection. Our study demonstrated that adding ECT to standard neoadjuvant treatment is feasible. A total of sixteen patients were enrolled in the study. ECT was generally well tolerated in our study population. In the ECT group, 86% of patients experienced post-ECT pain, which was predominantly transient and manageable. One device-related SAE was reported, involving a patient who experienced rectal pain requiring admission to the surgical ward 20 days post-ECT for pain management. Although the majority of patients treated with ECT do not report significant pain [36], previous radiotherapy has been found to be a significant predictor of post-procedure pain [36,37]. Interestingly, other studies on colorectal cancer using electroporation either for electrochemotherapy [17] or electroporation with calcium [38,39], which treated patients not previously treated with radiotherapy, did not report post-procedural pain. Furthermore, a study examining predictors of pain after ECT treatment of patients with cutaneous metastases showed that patients with pre-existing pain problems may experience exacerbation after ECT [36]. This could account for the high percentage of ECT patients in our study reporting rectal pain complaints after intervention, as all patients had undergone radiotherapy in the weeks leading up to ECT treatment and patients in the ECT group reported higher pain scores at baseline than patients in the control group. QoL measured by the EORTC QLQ C-30 questionnaire showed that ECT patients experienced reduced QoL following ECT treatment. However, in our study population this deterioration was temporary, and QoL was regained by 12 weeks post-surgery. Future studies should consider reversing the treatment order, administering ECT before radiotherapy and pre-treatment assessment of pain and implementation of individualised pain management strategies to help mitigate post-procedural pain. To the best of our knowledge, this was the first randomised controlled study to evaluate ECT as a neoadjuvant treatment modality in non-metastatic rectal cancer.

Our findings are consistent with a clinical trial investigating calcium electroporation as a bridging procedure prior to curative surgery for early rectal and sigmoid cancers, which reported few adverse events and demonstrated the feasibility of electroporation-based neoadjuvant treatment in CRC [38]. Notably, the same study also reported that the planned surgical resections were not compromised by the experimental calcium electroporation, with all patients achieving microscopic R0 resection. In contrast, our study observed iatrogenic tumour perforations during resection in two patients within the ECT group. This is a discouraging finding, as tumour perforation in rectal cancer is a negative predictor associated with an increased risk of recurrence [40,41]. Although the operating surgeons did not attribute the surgical complications in the ECT group directly to the neoadjuvant ECT treatment in our study, a contribution from ECT cannot be excluded. ECT treatment induces both local tissue oedema and necrosis, which may have contributed to a more challenging surgical field and the intraoperative complications observed. However, given the small sample size of the present study, no causal association between ECT and the observed perforations can be established. Our results suggest that adding ECT to standard neoadjuvant CRT is technically feasible. However, the present data do not establish that the combination is safe: ECT was associated with substantial post-procedural pain, and intra-operative perforation occurred in two of the six resected ECT patients (33%) compared with none of the control patients. The safety profile, particularly with respect to surgical complications, therefore needs to be explored in future adequately powered studies.

The multimodal study design and reduced sample size in our study preclude assessment of the isolated efficacy of ECT, and no comparative statistical analysis between the ECT and control groups was performed. Consequently, the present study cannot detect an association between ECT and tumour response. Only descriptive analyses were performed to illustrate tumour response, and the findings should be considered exploratory. A key limitation of our study is the small sample size, which prevented comparative statistical analysis between the ECT and control groups. In addition, missing outcome data, especially in the ECT group, was an issue. Assessment of tumour response was further complicated by treatment timing, as all patients received neoadjuvant CRT in the weeks leading up to intervention with ECT. The median time was 29 days between CRT completion and ECT, and 43 days between ECT and surgery. The median time between completed CRT and surgery was 69 days for both the control and intervention groups. As tumour regression following neoadjuvant CRT for LARC continues for several weeks after treatment completion, with most notable tumour regression occurring within 8–11 weeks [42,43,44], it is not possible to distinguish ECT’s isolated efficacy. Furthermore, PET-MRI was not found suitable for assessment of tumour response this early after completion of the multimodal neoadjuvant treatment in our study, mainly due to local fibrosis and inflammation. Therefore, the PET-MRI findings should be considered exploratory. Despite these limitations, the multimodal study design also represents a strength. The study demonstrates the feasibility of integrating ECT as an add-on to the current standard of care CRT regimen in the management of LARC. This is clinically relevant, as multimodal strategies have been shown to increase the proportion of patients achieving pathological complete response, which is strongly associated with improved survival outcomes [45]. In this context, ECT may represent a potential strategy to further enhance tumour regression when combined with CRT. However, its efficacy and safety profile within the neoadjuvant setting remain to be established in larger studies. Despite the limitations of the present study, the findings offer valuable clinical insights, especially regarding the need for pre-ECT pain assessment and sufficient pain management in patients with LARC, and highlight the importance of closely assessing surgical outcomes in future studies investigating neoadjuvant ECT. An ongoing phase IIb randomised controlled study is currently enrolling LARC patients to investigate whether ECT treatment in the intervention group may contribute to a higher rate of complete or near-complete response, potentially allowing a more conservative surgical approach with local excision of the tumour instead of total mesorectal excision [46]. Tumour response to neoadjuvant treatment has been shown to be a predictor of survival, with pathological complete response correlating with improved overall and disease-free survival as well as higher sphincter-preservation rates in rectal cancer [47,48]. Thus, treatment strategies that can increase the rate of complete response have the potential to improve patient survival outcomes and potentially also reduce treatment-related morbidity. There has been a paradigm shift in recent years toward adopting Total Neoadjuvant Therapy (TNT), with non-operative management being considered for patients achieving cCR. In this context, the potential role of ECT warrants further investigation. If future studies demonstrate efficacy and establish ECT as a safe treatment option in LARC, ECT could potentially be investigated as an adjunct to TNT, either as induction or consolidation therapy, with the aim of increasing tumour response and the proportion of patients achieving cCR. Furthermore, patients achieving cCR may subsequently be considered for a watch-and-wait strategy, avoiding the morbidity associated with surgical resection. However, whether ECT can safely and effectively contribute to TNT and increase the proportion of patients eligible for non-operative management remains to be established.

Beyond its cytotoxic effects, ECT may also induce immunostimulatory effects, which have been suggested by preclinical studies in murine models [49,50,51,52]. However, this has yet to be explored in clinical studies investigating CRC. As part of this trial, biological material was collected for a future translational study to assess immune modulation following ECT in LARC.

## 5. Conclusions

Electrochemotherapy is technically feasible as an adjunct to neoadjuvant chemoradiotherapy in the treatment of locally advanced rectal cancer. However, its efficacy and safety profile, particularly with regard to surgical complications, remains unclear. The occurrence of iatrogenic perforations during surgery following ECT highlights the need for careful evaluation of surgical outcomes in future studies. The observed post-procedural pain also emphasises the importance of pre-ECT pain assessment and management. Whether ECT can be safely incorporated in the multimodal neoadjuvant management of LARC in the future remains to be investigated in future trials.

## Figures and Tables

**Table 1:** Patient characteristics.

Characteristic	Overall, *N* = 16	Control, *N* = 8	ECT, *N* = 8
Gender			
Male	10 (63%)	5 (63%)	5 (63%)
Female	6 (38%)	3 (38%)	3 (38%)
Age ^1^	59 (50, 67)	66 (46, 69)	54 (52, 62)
T-stage at study entry			
3	13 (81%)	5 (63%)	8 (100%)
4	3 (19%)	3 (38%)	0 (0%)
N-stage at study entry			
0	6 (38%)	2 (25%)	4 (50%)
1	4 (25%)	1 (13%)	3 (38%)
2	6 (38%)	5 (63%)	1 (13%)
M-stage at study entry	0 (0%)	0 (0%)	0 (0%)
ASA			
1	9 (56%)	4 (50%)	5 (63%)
2	4 (25%)	1 (13%)	3 (38%)
3	3 (19%)	3 (38%)	0 (0%)
WHO Performance Status			
0	14 (88%)	6 (75%)	8 (100%)
1	2 (13%)	2 (25%)	0 (0%)
Primary histological diagnosis			
Adenocarcinoma	13 (81%)	7 (88%)	6 (75%)
Glandular adenocarcinoma	1 (6%)	0 (0%)	1 (13%)
Mucinous adenocarcinoma	2 (13%)	1 (13%)	1 (13%)
UICC at study entry			
2	6 (38%)	2 (25%)	4 (50%)
3	10 (63%)	6 (75%)	4 (50%)
Tumour distance from anal verge			
<5 cm	10 (63%)	5 (63%)	5 (63%)
6–10 cm	6 (38%)	3 (38%)	3 (38%)
Tumour volume on MRI, cm^3^			
Tumour volume after neoadjuvant CRT ^1^	18.8 (11.3, 32.8)	21.2 (13.7, 33.8)	13.6 (12.7, 17.3)
Missing data	4 (25%)	1 (13%)	3 (38%)

Due to rounding, some fields add up to 101% instead of 100%. *n* (%); ^1^ median (interquartile range).

**Table 2:** Surgery characteristics.

Characteristic	Overall, *N* = 14	Control, *N* = 8	ECT, *N* = 6
Total procedure time (min) ^1^	269 (175–390)	274 (178–376)	269 (175–390)
Missing	5	2	3
Blood loss (mL) ^1^	75 (0–290)	50 (0–100)	100 (0–290)
Missing	4	3	1
Surgical procedure			
Low anterior resection	3 (21%)	3 (38%)	0
Abdominoperineal resection	5 (36%)	2 (25%)	3 (50%)
Extralevator dissection	4 (29%)	3 (38%)	1 (17%)
TaTME	2 (14%)	0	2 (33%)
Surgical modality			
Laparoscopic	7 (50%)	2 (25%)	5 (83%)
Robotic	4 (29%)	4 (50%)	0
Open	3 (21%)	2 (25%)	1 (17%)
Conversion			
No	14 (100%)	8 (100%)	6 (100%)
Macroscopic R0 resection			
No	2 (14%)	0 (0%)	2 (33%)
Yes	12 (86%)	8 (100%)	4 (67%)
Postoperative complication			
Clavien–Dindo 1	2 (14%)	1 (13%)	1 (17%)
Clavien–Dindo 3	3 (21%)	2 (25%)	1 (17%)
No postoperative complication	9 (64%)	5 (63%)	4 (67%)
Length of stay ^1^	7 (3–35)	7 (3–31)	6 (5–35)

Due to rounding, some fields add up to 99% or 101% instead of 100%. *n* (%); ^1^ median (range).

## Data Availability

The data presented in this study are available upon reasonable request from the corresponding author. The data are not publicly available due to privacy and legal restrictions.

## Supplementary Materials

The following supporting information can be downloaded at https://www.mdpi.com/article/10.3390/cancers18183066/s1, Supplementary Table S1a: Safety outcomes. Common Terminology Criteria for Adverse Events version 4.0. Supplementary Table S1b: AEs/SAEs in the electrochemotherapy group. Supplementary Table S2a: Individual patient tumour staging, treatment response, and long-term recurrence status. Electrochemotherapy group. Supplementary Table S2b: Individual patient tumour staging, treatment response, and long-term recurrence status. Control group. Supplementary Figure S1: (a) and (b) European Organisation for Research and Treatment of Cancer Quality of Life Questionnaires Core. Function scales in the ECT and control groups. A higher score indicates better health-related quality of life; (c) and (d) European Organisation for Research and Treatment of Cancer Quality of Life Questionnaires Core. Symptom scales in the ECT and control groups. A higher score indicates worse health-related quality of life. Supplementary Table S3a: European Organisation for Research and Treatment of Cancer Quality of Life Questionnaires Core mean score for the ECT group. * A higher score indicates worse health-related quality of life. ** A higher score indicates better health-related quality of life. Supplementary Table S3b: European Organisation for Research and Treatment of Cancer Quality of Life Questionnaires Core mean score for the control group. Supplementary Table S4: 11-point Pain Intensity Numeric Rating Scale, where 0 = no pain and 10 = worst possible pain. Median (range). Supplementary Figure S2: Low Anterior Resection Syndrome total score over time by treatment group. Major LARS score 30–42, minor LARS score 21–29, no LARS score 0–20. Supplementary Figure S3a: Male Lower Urinary Tract Symptoms scores over time by treatment group. A high score indicates worse functioning. Supplementary Figure S3b: Female Lower Urinary Tract Symptoms scores over time by treatment group. A high score indicates worse functioning. Supplementary Table S5: International Index of Erectile Function. Results for sexually active respondents only. A high score indicates better function. Medians (range). Supplementary Table S6: Sexual Function Vaginal Changes Questionnaire. Results for sexually active respondents only. A high score indicates better function, except in the vaginal changes domain, which indicates more symptoms related to worse vaginal functioning. Medians (range).

## Abbreviations

The following abbreviations are used in this manuscript.

CRC	Colorectal cancer
LARC	Locally advanced rectal cancer
CRT	Chemoradiotherapy
cCR	Clinical complete response
ECT	Electrochemotherapy
MDT	Multidisciplinary team
ICD	Implantable cardioverter defibrillator
PET	Positron emission tomography
MRI	Magnetic resonance imaging
ECG	Electrocardiography
QoL	Quality of life
NAPS	Nurse-assisted propofol sedation
PEF	Pulsed Electric Fields
TRG	Mandard tumour regression grade
AE	Adverse event
SAE	Serious adverse event
CTCAE	Common Terminology Criteria for Adverse Events
EORTC QLQ-C30	European Organisation for Research and Treatment of Cancer Quality of Life Questionnaire Core 30
NRS	11-point Pain Intensity Numeric Rating Scale
LARS	Low Anterior Resection Syndrome
ICIQ-MLUTS	International Consultation on Incontinence Questionnaire Male Lower Urinary Tract Symptoms Module
ICIQ-FLUTS	International Consultation on Incontinence Questionnaire Female Lower Urinary Tract Symptoms Module
IIEF	International Index of Erectile Function
SVQ	Sexual Function Vaginal Changes Questionnaire
FDG	Flouro-deoxy-glucose
ADC	Apparent diffusion coefficient
SUV	Standardised Uptake Value
CRF	Case report form
ASA	American Society of Anaesthesiologists
TNT	Total Neoadjuvant Therapy
