# Supplementary material for: Safety and Feasibility of Endoscopic-Assisted Electrochemotherapy in Addition to Neoadjuvant Treatment of Locally Advanced Rectal Cancer: An Exploratory Randomised Clinical Trial
Source: Cancers (Basel). 2026 Sep 21;18(18):3066. doi: 10.3390/cancers18183066 (PMC13605616; doi:10.3390/cancers18183066)
Supplement: Supplementary file 1 [file cancers-18-03066-s001.zip › cancers-4446676-supplementary.pdf]

Adverse event	ECT, N = 7	Control, N = 8
Any AEs	7 (100%)	4 (50%)
SAEs	2 (29%)	0
Bleomycin-related AEs	1 (14%)	
Electroporation-related AEs	6 (86%)	
Bleomycin-related SAEs	0	
Electroporation-related SAEs	1 (14%)	

Supplementary Table S1a. Safety outcomes. Common Terminology Criteria for Adverse Events version 4.0.

Patient no.	AEs	CTCAE-grading	Related to study drug/device
2	Nausea	1	Y
	Rectal pain	2	Y
	Rectal ulcer	2	Y
	Bloating	2	Y
	Colonic obstruction	2	Y
	Constipation	2	Y
	Fever	1	Y
	Fatigue	1	Y
5	Rectal pain	2	Y
	Constipation	2	Y
	Urinary retention	1	N
	Abdominal pain	1	Y
	Fall	3	N
	Nausea	2	Y
6	Diarrhea	2	Y
	Faecal incontinence	2	Y
	Rectal pain	2	Y
	Urinary retention	2	N
8	Rectal pain	2	Y
	Proctitis	2	Y
9	Rectal pain	2	Y
	Rectal ulcer	2	Y
	Frequent urge to pass stool	2	Y
10	Perineal wound	2	N

11	Rectal pain	3	Y
	Urinary retention	2	N
	Fatigue	3	N

Supplementary Table S1b. AEs/SAEs in the electrochemotherapy group.

Patient no.	ycTNM	ypTNM	Tumour regression grading	PET, tumour response (%) SUVmax	MRI, tumour size change (%)	Recurrence status (last follow-up April 2023)
2	cT3N0M0	ypT3N0M0	4	-5.26	277.78	No recurrence
5	cT3N0M0	ypT0N0M0	1	0	179.41	No recurrence
6	cT3N1M0	ypT3N0M0	3	n/a	n/a	No recurrence
8	cT3N0M0	ypT0N0M0	1	-20.43	-93.70	No recurrence
9	cT3N1M0	no resection – watchfull waiting	n/a	-100	-100	No recurrence, last colonoscopy in November 2022
10	cT3N2M0	ypT2N0M0	2	n/a	n/a	Recurrence with metastases to lungs and spine in June 2019, dies in September 2020.
11	cT3N0M0	no resection – metastatic disease	n/a	n/a	n/a	Recurrence. Lung- and liver metastases, diagnosed in October 2019 and receives palliative treatment. Dies in December 2019.
16	cT3N1M0	ypT3N0M0	3	17.08	-25.64	Local recurrence in April 2020. Dissemination to lungs, mediastinum and iliaca glands, diagnosed in September 2020. Dies April 2023.

Supplementary Table S2a. Individual patient tumour staging, treatment response, and long-term recurrence status. Electrochemotherapy group.

Patient no.	cTNM	ypTNM	Tumour regression grading	PET, tumour response (%) SUVmax	MRI, tumour size change (%)	Recurrence status (last follow-up April 2023)
1	cT3N2M0	ypT0N0M0	1	-100	-41.67	No recurrence
3	cT4N2M0	ypT3N1M0	5	16.31	-19.61	No recurrence
4	cT4N2M0	ypT4N1M0	3	0.71	-52.04	Recurrence with lung metastases in November 2018, dies March 2019.
7	cT4N0M0	ypT2N0M0	2	1.47	-100	No recurrence
12	cT3N2M0	ypT4N1M0	3	60.57	18.87	No recurrence
13	cT3N1M0	ypT3N2M0	4	-61.16	8.18	Recurrence with lymph node metastasis in September 2019, lung metastases in May 2021. Dies in January 2023.
14	cT3N2M0	ypT3N2M0	2	-100	-29.70	No recurrence
15	cT3N0M0	ypT0N0M0	1	n/a	n/a	Recurrence in September 2019, emergency admission with ileus, laparotomy with peroperative findings of carcinomatosis on small bowel. Dies September 2019.

Supplementary Table S2b. Individual patient tumour staging, treatment response, and long-term recurrence status. Control group.

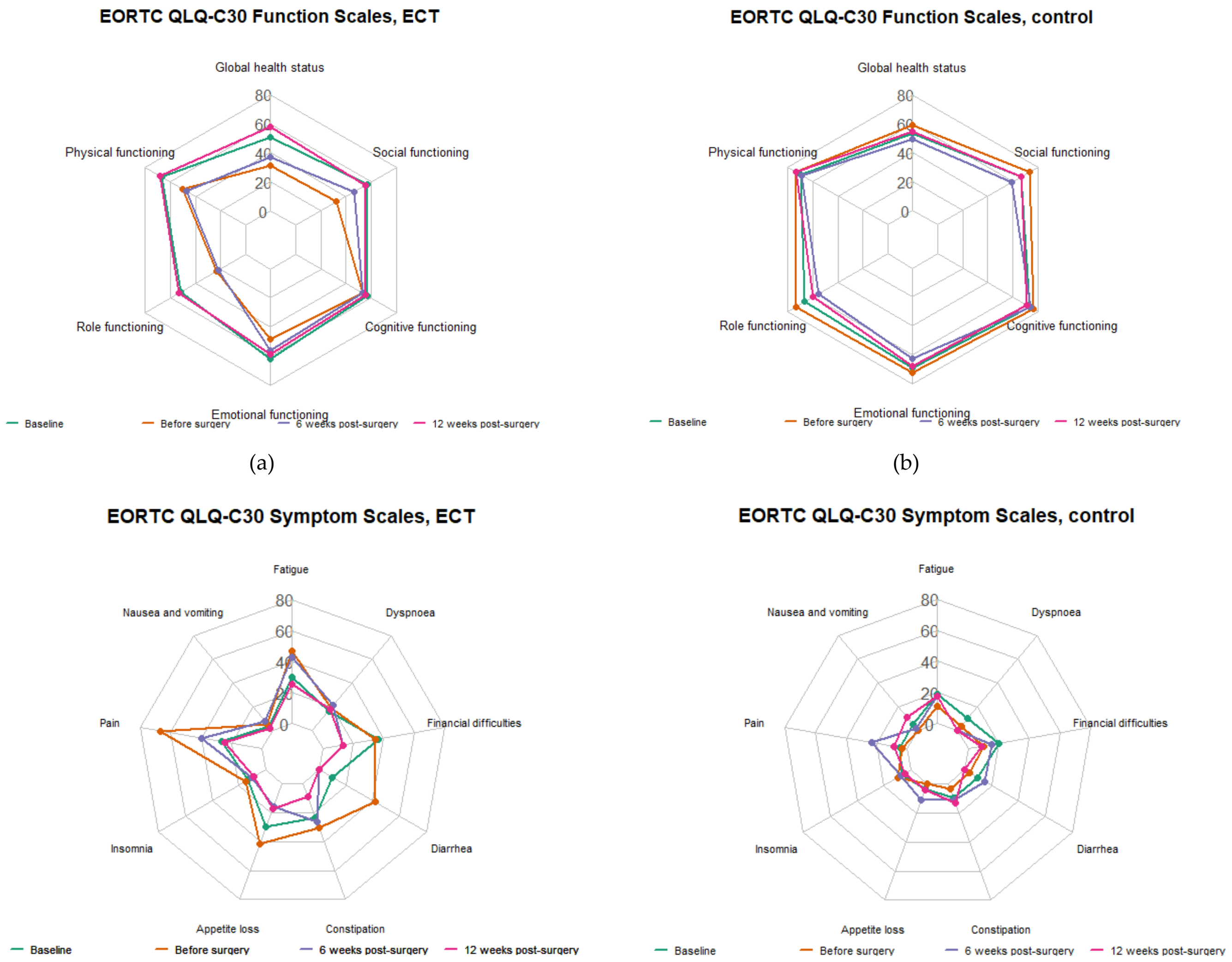

(a) (b) (c) (d)

Supplementary Figure S1. (a) and (b) European Organization for Research and Treatment of Cancer Quality of Life Questionnaires Core. Function scales in ECT and control group. A higher score indicates better health-related quality of life. (c) and (d) European Organization for Research and Treatment of Cancer Quality of Life Questionnaires Core. Symptom scales in the ECT and control group. A higher score indicates worse health-related quality of life.

Domain	Baseline (n = 8)	Pre-op (n = 7)	6 weeks post-op (n = 5)	12 weeks post-op (n = 6)
**Global health status****	63.5	39.3	46.7	72.2
**Functional scales****				
**Physical functioning**	83.3	62.9	59.0	85.6
**Role functioning**	64.6	28.6	26.7	66.7
**Emotional functioning**	77.1	60.7	70.0	73.6
**Cognitive functioning**	70.8	66.7	66.7	69.4
**Social functioning**	70.8	40.5	58.3	69.4
**Symptom scales***				
**Fatigue**	37.5	58.7	53.3	31.5
**Nausea and vomiting**	4.2	7.1	10.0	2.8
**Pain**	33.3	83.3	50.0	30.6
**Dyspnoea**	16.7	19.0	13.3	11.1
**Insomnia**	37.5	52.4	20.0	22.2
**Appetite loss**	29.2	38.1	33.3	11.1
**Constipation**	12.5	52.4	0.0	0.0
**Diarrhea**	45.8	42.9	16.7	16.7
**Financial difficulties**	20.8	23.8	26.7	22.2

Supplementary Table S3a. European Organization for Research and Treatment of Cancer Quality of Life Questionnaires Core mean score for the ECT group. *A higher score indicates worse health-related quality of life. **A higher score indicates better health-related quality of life.

Domain	Baseline (n = 8)	Pre-op (n = 8)	6 weeks post-op (n = 7)	12 weeks post-op (n = 6)
**Global health status****	66.7	74.0	61.9	68.1
**Functional scales****				
**Physical functioning**	87.5	91.7	85.7	92.2
**Role functioning**	83.3	91.7	69.0	75.0
**Emotional functioning**	86.5	90.6	78.6	84.7
**Cognitive functioning**	91.7	95.8	92.9	88.9
**Social functioning**	83.3	91.7	73.8	83.3
**Symptom scales***				
**Fatigue**	23.6	13.9	22.2	22.2
**Nausea and vomiting**	6.2	0.0	2.4	13.9
**Pain**	6.2	4.2	28.6	11.1
**Dyspnoea**	8.3	12.5	9.5	5.6
**Insomnia**	4.2	0.0	14.3	5.6
**Appetite loss**	12.5	4.2	14.3	16.7
**Constipation**	12.5	4.2	19.0	0.0
**Diarrhea**	25.0	12.5	19.0	11.1
**Financial difficulties**	12.5	4.2	0.0	0.0

Supplementary Table S3b. European Organization for Research and Treatment of Cancer Quality of Life Questionnaires Core mean score for the control group.

	ECT				control			
	Baseline (n = 8)	Before surgery (n = 7)	6 weeks post-surgery (n = 4)	12 weeks post-surgery (n =6)	Baseline (n =8)	Before surgery (n =8)	6 weeks post-surgery (n =5)	12 weeks post-surgery (n =6)
**Current pain**	1.5 (0-7)	6 (4-7)	4 (2-6)	0.5 (0-8)	0 (0-5)	0 (0-1)	0 (0-6)	0 (0-1)
**Worst pain during the last 7 days**	5 (0-9)	8 (7-9)	6.5 (3-9)	1.5 (0-8)	1 (0-5)	0.5 (0-5)	3 (0-8)	1.5 (0-5)
**Average pain during the last 7 days**	2.5 (0-6)	7 (4-9)	4.5 (1-7)	0.5 (0-8)	1 (0-5)	0.5 (0-3)	1 (0-7)	0.5 (0-5)

Supplementary Table S4. 11-point Pain Intensity Numeric Rating Scale, where 0=no pain and 10=worst possible pain. Median (range).

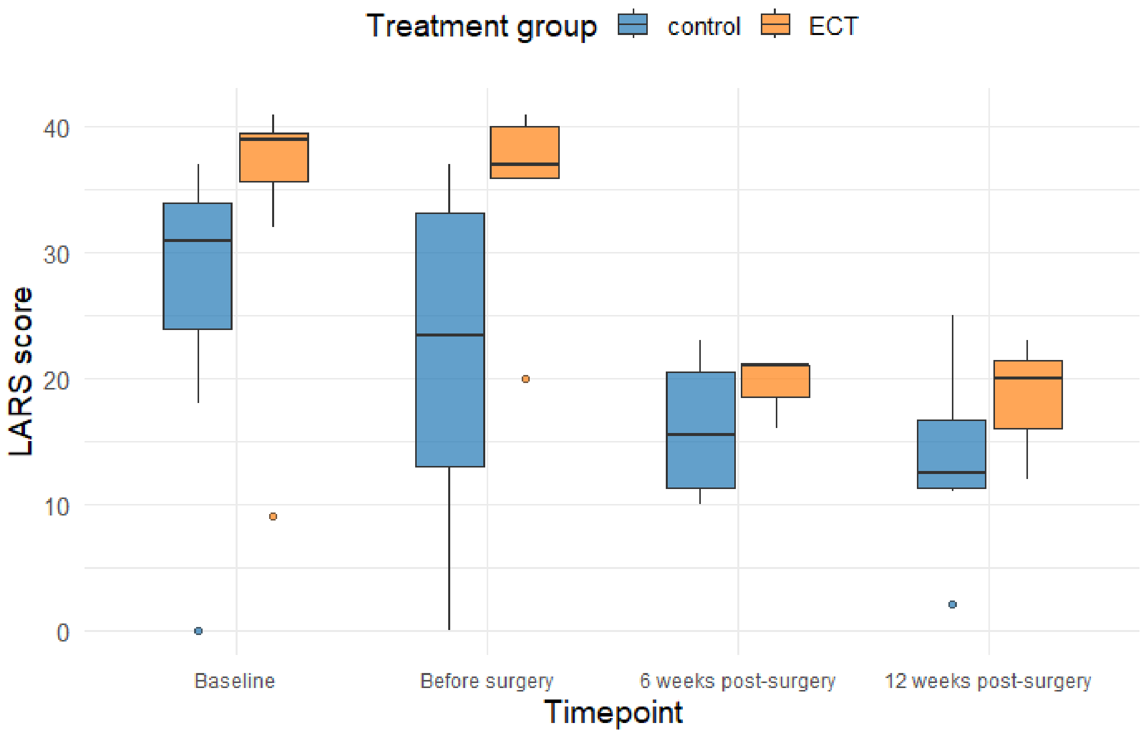

Supplementary Figure S2. Low Anterior Resection Syndrome total score over time by treatment group. Major LARS score 30-42, minor LARS score 21-29, no LARS score 0-20.

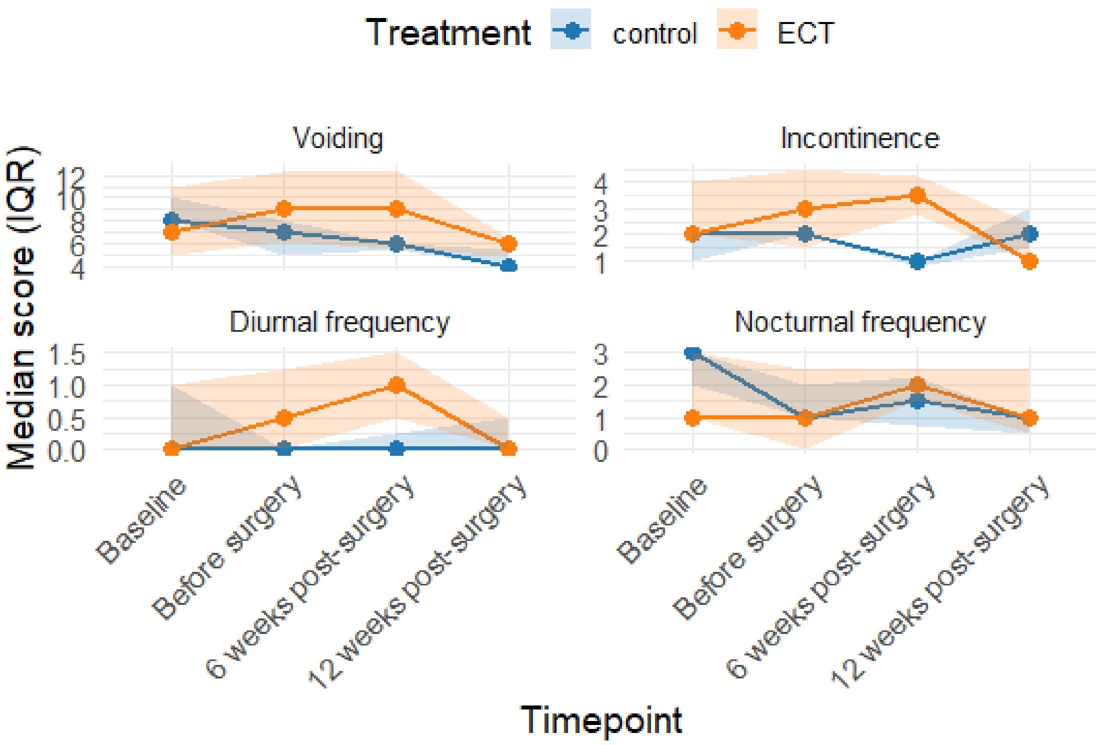

Supplementary Figure S3a. Male Lower Urinary Tract Symptoms scores over time by treatment group. A high score indicates worse functioning.

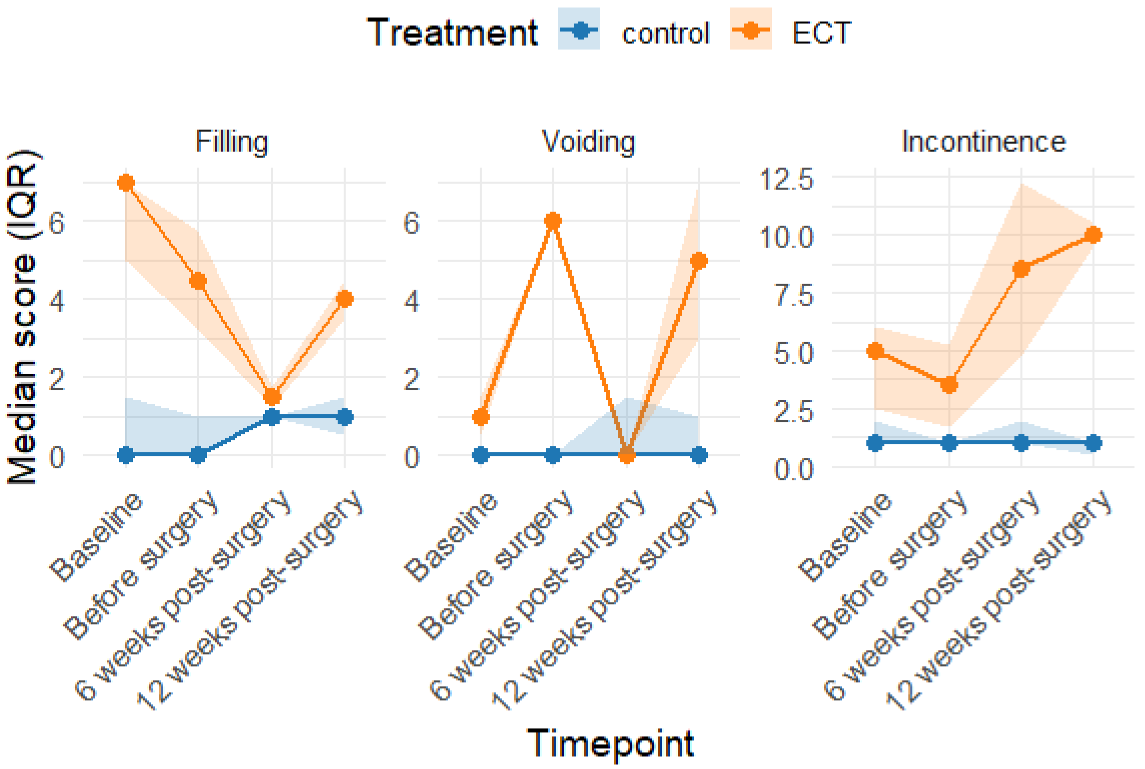

Supplementary Figure S3b. Female Lower Urinary Tract Symptoms scores over time by treatment group. A high score indicates worse functioning.

	ECT				control			
	Baseline (n = 4)	Before surgery (n = 3-4)	6 weeks post-surgery (n = 1)	12 weeks post-surgery (n = 2)	Baseline (n = 3)	Before surgery (n = 3)	6 weeks post-surgery (n = 2)	12 weeks post-surgery (n = 3)
**Erectile function**	13 (11-30)	11.5 (4-24)	3 (3-3)	11 (9-13)	28 (28-29)	10 (3-11)	9 (9-9)	12 (8-12)
**Orgasmic function**	8.5 (4-10)	0 (0-2)	2 (2-2)	2 (2-2)	10 (6-10)	2 (2-6)	2 (2-2)	2 (2-2)
**Sexual desire**	6 (4-8)	5 (2-6)	5 (5-5)	4 (4-4)	7 (7-7)	3 (3-3)	3.5 (2-5)	4 (2-5)
**Intercourse satisfaction**	3 (0-12)	0 (0-4)	0 (0-0)	5 (5-5)	11 (11-12)	4 (0-5)	2.5 (0-5)	4 (0-5)
**Overall satisfaction**	5 (3.5-7)	6 (5-8)	4 (4-4)	2 (0-4)	8 (6-8)	2 (2-7)	4 (4-4)	4 (3-5)

Supplementary Table S5. International Index of Erectile Function. Results for sexually active respondents only. A high score indicates better function. Medians (range).

	ECT				control			
	Baseline (n =2-3)	Before surgery (n =0-1)	6 weeks post-surgery (n =1-2)	12 weeks post-surgery (n = 2)	Baseline (n = 1-2)	Before surgery (n = 1-2)	6 weeks post-surgery (n = 1)	12 weeks post-surgery (n = 1)
**Intimacy**	6 (5-8)	6 (6-6)	6 (6-6)	6.5 (6-7)	6 (6-6)	6.5 (6-7)	8 (8-8)	6 (6-6)
**Sexual interest**	3 (3-4)	2 (2-2)	2 (1-3)	2.5 (2-3)	2 (2-2)	3 (3-3)	2 (2-2)	2 (2-2)
**Global sexual satisfaction**	5 (4-6)	5 (5-5)	5 (4-6)	6.5 (6-7)	6 (5-7)	7.5 (7-8)	4 (4-4)	7 (7-7)
**Vaginal changes**	11.5 (10-13)	NA	14 (14-14)	15.2 (9.3-21)	13 (13-13)	5.3 (5.3-5.3)	8 (8-8)	7 (7-7)
**Sexual functioning**	7 (5-9)	NA	6 (6-6)	8 (8-8)	7 (7-7)	9 (9-9)	5 (5-5)	5 (5-5)

Supplementary Table S6. Sexual Function Vaginal Changes Questionnaire. Results for sexually active respondents only. A high score indicates better function, except in the vaginal changes domain which indicates more symptoms related to worse vaginal functioning. Medians (range).
